# Taraxasterol Acetate Attenuates TNF-α-Induced Insulin Resistance via Regulation of Insulin Signaling, Inflammation, and Lipid Metabolism in 3T3-L1 Cells

**DOI:** 10.1021/acsomega.5c12241

**Published:** 2026-02-26

**Authors:** Renan P. de Lima, Francisca Tuelly B. de Oliveira, Ana Virginia L. da Silva, Maria Rose Jane R. Albuquerque, Otília D. L. Pessoa, Flávia A. Santos

**Affiliations:** † Department of Medicine, Weill Center for Metabolic Health, Cardiovascular Research Institute, 573865 Weill Cornell Medicine, New York, New York 10021, United States; ‡ Department of Physiology and Pharmacology, 28121 Federal University of Ceará, Fortaleza, Ceará 60430-270, Brazil; § 307195 Vale do Acaraú State University, Sobral, Ceará 62010-295, Brazil; ∥ Department of Organic and Inorganic Chemistry, Sciences Center, Federal University of Ceará, Fortaleza, Ceará 60440-900, Brazil

## Abstract

Insulin resistance, obesity, and type 2 diabetes mellitus (T2DM) are interrelated metabolic disorders with rising global prevalence. Triterpenes, known for their diverse pharmacological properties, have shown potential in improving insulin sensitivity and exerting antidiabetic and antiobesity effects. This study evaluated the effects of taraxasterol acetate (TXA), a pentacyclic triterpene isolated from *Eupatorium ballotaefolium*, on TNF-α-induced insulin resistance and lipolysis in mature 3T3-L1 adipocytes. TXA significantly enhanced glucose uptake in insulin-resistant adipocytes by promoting GLUT4 translocation by activating the IRS-1/PI3K/Akt signaling pathway and upregulating AMPK expression. TXA also inhibited NF-κB and JNK signaling, reducing inflammation and mitigated oxidative stress by decreasing intracellular reactive oxygen species (ROS) levels and enhancing antioxidant enzyme activity, including superoxide dismutase (SOD) and catalase (CAT). Moreover, TXA normalized adipokine secretion by increasing leptin and adiponectin levels, and promoted lipid accumulation through the modulation of PPARγ, HSL, ATGL, and Perilipin expression. TXA further improved lipid metabolism by upregulating fatty acid β-oxidation genes (ACOX1, CPT1b) and supported mitochondrial function by enhancing PGC-1α and TFAM expression. Collectively, these findings demonstrate that TXA mitigates TNF-α-induced insulin resistance by improving insulin signaling, suppressing inflammation and oxidative stress, and improving lipid and mitochondrial metabolism. These results suggest that TXA is a promising therapeutic candidate for the prevention and treatment of insulin resistance and related metabolic disorders.

## Graphical abstract

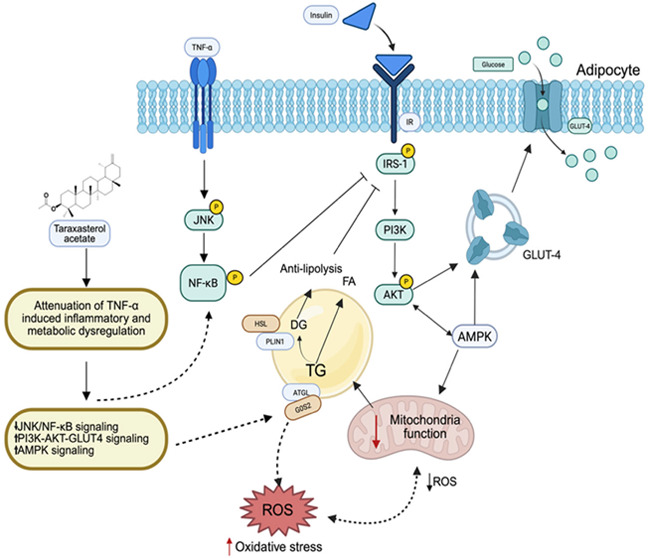

## 1 Introduction

The global prevalence of obesity, type 2 diabetes mellitus (T2DM), and insulin resistance has risen dramatically in recent decades, making these interconnected metabolic disorders major public health concerns.^1^ Obesity is a central contributor to metabolic dysfunction and is strongly associated wtih insulin resistance, T2DM, cardiovascular diseases, and certain types of cancer.^2^ Since 1975, the global prevalence of obesity has more than tripled, serving as a key driver of the rising burden of T2DM.^3^

Chronic overnutrition leads to excessive fat accumulation in adipose tissue, resulting in adipocyte hypertrophy and hyperplasia.^4^ These changes promote tissue hypoxia, immune cell activation, dysregulated adipokine secretion, and persistent low-grade inflammation. These factors collectively promote insulin resistance and play a critical role in the development of obesity and T2DM.^5^

Among pro-inflammatory mediators, tumor necrosis factor-α (TNF-α) plays a pivotal role in regulating energy metabolism and inflammation in adipose tissue.^6^ TNF-α induces insulin resistance by activating serine/threonine kinases, including the mitogen-activated protein kinases (MAPKs) such as extracellular signal-regulated kinases 1/2 (ERK1/2), p38, and c-Jun N-terminal kinase (JNK), and by regulating the nuclear factor kappa B (NF-κB) pathway. These signaling events lead to insulin receptor substrate 1 (IRS-1) phosphorylation, impaired phosphatidylinositol-3-kinase (PI3K)/protein kinase B (PKB/Akt) signaling and GLUT4 translocation.^6–9^ TNF-α also downregulates metabolic proteins like IRS-1, GLUT4, peroxisome proliferator-activated receptor-γ (PPARγ), and perilipin, contributing to impaired glucose metabolism. Additionally, TNF-α enhances lipolysis by blocking insulin’s antilipolytic effects, stimulating triglyceride hydrolysis, inhibiting adenosine receptor signaling,^6,10^ and directly acting on perilipin.^11^ It disrupts energy homeostasis by upregulating protein phosphatase 2C, inhibiting AMP-activated protein kinase (AMPK), suppressing fatty acid oxidation, increasing diacylglycerol accumulation, and promoting insulin resistance.^12,13^

Conventional treatments for insulin resistance include lifestyle interventions and pharmacological agents such as metformin and thiazolidinediones. However, their clinical application is often limited by adverse effects, including gastrointestinal discomfort, fluid retention, and increased fracture risk.^14,15^ This has spurred growing interest in identifying safer, plant-derived bioactive compounds.

Natural pentacyclic triterpenes represent a promising class of therapeutic agents due to their ability to regulate transcription factors, protein kinases, and metabolic enzymes involved in insulin resistance.^16^ Compounds such as oleanolic acid, ursolic acid, α,β-amyrin, and lupeol have been shown to enhance insulin sensitivity, improve glucose and lipid metabolism and exhibit low toxicity in preclinical studies.^16^ In particular, ursane-type triterpenoids like α-amyrin and ursolic acid have been reported to stimulate glucose uptake and reduce free fatty acid (FFA) production by upregulating the Akt-GLUT4 pathway and PPARγ in adipocytes.^17,18^ Taraxasterol, a pentacyclic triterpene first isolated from *Taraxacum officinale*, has been shown to activate key components of the insulin signaling pathway, including p-IRS-1, p-AKT, and p-GSK-3β, in a model of aging cardiomyocytes not directly related to diabetes, suggesting a potential role in improving insulin sensitivity.^19^

Taraxasterol acetate (TXA) (Figure 1A) is a naturally occurring ursane-type pentacyclic triterpene isolated from *Eupatorium ballotifolium* Kunth (Asteraceae).^20^ TXA has demonstrated hepatoprotective,^21^ anti-inflammatory,^22,23^ and anticancer activities.^24^ While other pentacyclic triterpenes such as oleanolic acid, ursolic acid, and α,β-amyrin have shown efficacy in improving glucose uptake and insulin secretion,^25–27^ the metabolic effects of TXA’s, particularly in the context of insulin resistance, remain unexplored. This study aims to investigate the effects of TXA on TNF-α-induced insulin resistance and lipolysis in 3T3-L1 adipocytes. We evaluate its impact on insulin signaling, inflammation, oxidative stress, lipid metabolism and mitochondrial function to assess its therapeutic potential for metabolic disorders such as obesity and T2DM.

**Figure 1:**
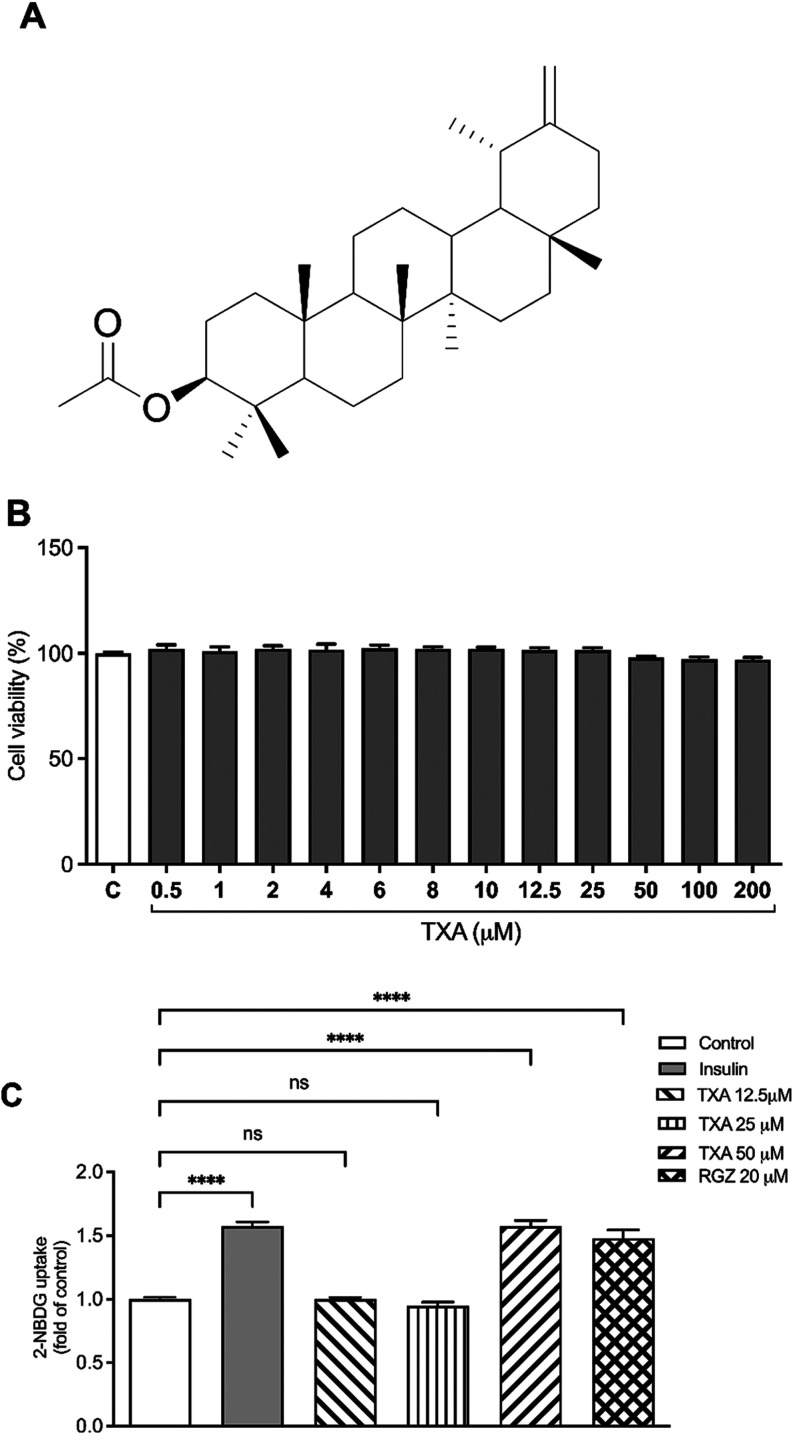
Taraxasterol acetate (TXA) ameliorates glucose uptake in adipocytes. (A) Chemical structure of taraxasterol acetate (TXA). (B) Cell viability was assessed using the MTT assay. (C) TXA increases glucose uptake in adipocytes without affecting cell viability. Glucose uptake was measured using the 2-NBDG assay. The data are expressed as the mean ± SD (*n* = 3). ****p < 0.0001 (ANOVA followed by Tukey test).

## 2 Materials and Methods

### 2.1 Chemicals and Reagents

3-(4, 5-dimethylthiazol-2-yl)-2, 5-diphenyltetrazolium bromide (MTT), Dulbecco’s Modified Eagle’s Media (DMEM), fetal bovine serum (FBS), 4-(2-hydroxyethyl)­piperazine-1-ethanesulfonic acid (HEPES), 2-(*N*-(7-nitrobenz-2-oxa-1,3-diazol-4-yl)­Amino)-2-Deoxyglucose (2-NBDG), high-capacity cDNA reverse transcription kit (ThermoFisher, Waltham, MA, EUA), newborn calf serum (NBCS), penicillin-streptomycin, rosiglitazone, and other culture solutions and supplements were purchased from Gibco by Life Technologies (Waltham, MA, EUA). Dexamethasone, 2′,7′-dichlorodihydrofluorescein diacetate (DCFH-DA), 5,5′-dithiobis­(2-nitrobenzoic acid), dimethyl sulfoxide (DMSO), insulin, 3-isobutyl-1 methylxanthine (IBMX), Oil Red O, phenylmethylsulfonyl fluoride, protease inhibitor cocktail, Radioimmunoprecipitation assay buffer (RIPA) lysis buffer, sodium orthovanadate, thiobarbituric acid and TNF-α were obtained from Sigma-Aldrich (St Louis, MO, USA). QIAzol reagent was obtained from Qiagen (Hilden, Germany). The GoTaq qPCR master mix kit was obtained from Promega (Madison, WI, USA). Anti-AMPK (#2532), anti-pAMPK (Thr 172, #2531), anti-AKT (#9272), anti-pAKT (Ser 473, #9271), anti-PI3K (#4292), anti-IRS (#2382), anti-pIRS (Ser 307, #2381), anti-JNK (#9252), anti-pJNK (Thr 183/Tyr 185, #4668), anti-NF-κB (#8242), anti-pNF-κB (Ser 536, #3033) and anti-IgG HRP (#7076) antibodies were purchased from Cell Signaling (Danvers, MA, USA). Anti-GLUT4 (#sc-53566) and anti-β-actin (#sc-47778) monoclonal antibodies were purchased from Santa Cruz Biotechnology (Dallas, TX, USA). All other chemicals and solvents used were purchased from Sigma-Aldrich.

### 2.2 Taraxasterol Acetate Isolation

The aerial parts of *Eupatorium ballotaefolium* were collected at Meruoca, State of Ceará, Brazil. The plant material was identified by Dr. Edson de Paula Nunes, a botanist from the Federal University of Ceará (UFC). A voucher specimen (27,646) was deposited at the Herbarium Prisco Bezerra of the UFC. This work was registered in Brazil’s National System for Management of Genetic Heritage and Associated Traditional Knowledge (SisGen, no. ACB11B8).

Dried and powdered leaves of *E. ballotaefolium* (1.3 kg) were macerated with hexane at room temperature. The solvent was evaporated under reduced pressure to yield a crude hexane extract (21.6 g), which was subjected to a silica gel column eluted with petroleum ether, hexane, dichloromethane, and ethyl acetate. During petroleum ether evaporation, an impure compound was formed in significant amounts. When recrystallized from hot acetone twice, this material gave colorless crystals (1.5 g). Its structure was determined as taraxasterol acetate (TXA) (Figure 1A) by Fourier Transform Infrared (FT-IR, PerkinElmer, USA), Mass Spectrometry (MS, HP-5971AHewlett-Packard, USA), and Nuclear Magnetic Resonance (NMR, Bruker, USA) (Figures S1–S5, Supporting Information). IR, MS, and ^13^C NMR data for TXA, a pentacyclic triterpene of molecular formula C_32_H_52_O_2_, are described below.

#### 2.2.1 Taraxasterol Acetate

mp 220.1–221.6 °C; IR: 2941, 1731,1592, 1373, 1245, and 1026 cm^–1^; MS (70 eV) *m*/*z* 468; ^13^C NMR (125 MHz, CDCl_3_): 38.5 (C-1), 23.7 (C-2), 81.0 (C-3), 37.8 (C-4), 55.5 (C-5), 18.2 (C-6), 34.0 (C-7), 40.9 (C-8), 50.4 (C-9), 37.1 (C-10), 21.5 (C-11), 26.2 (C-12), 39.2 (C-13), 42.0 (C-14), 26.7 (C-15), 38.3 (C-16), 34.5 (C-17), 48.7 (C-18), 39.4 (C-19), 154.6 (C-20), 25.6 (C-21), 38.9 (C-22), 27.9 (C-23), 16.5 (C-24), 16.3 (C-25), 15.9 (C-26), 14.7 (C-27), 19.5 (C-28), 25.5 (C-29), 107.1 (C-30), CH_3_CO (21.3, 170.9).

### 2.3 Cell Culture and Differentiation

Murine 3T3-L1 preadipocyte cells were purchased from the American Type Culture Collection (ATCC, Manassas, VA, USA). Preadipocytes were cultured and differentiated into adipocytes as described previously.^28^ Cells were maintained in DMEM supplemented with 10% NBCS, 100 U/mL penicillin, and 0.1 μg/mL streptomycin at 37 °C under 5% CO_2_ atmosphere.

To induce adipocyte differentiation, cells were seeded at a density of 1 × 10^5^ cells/well in 12-well plates. Differentiation was then initiated (designated as “day 0”) in DMEM containing 10% FBS, 0.5 mM IBMX, 0.25 μM dexamethasone, 1 μg/mL insulin, 100 U/mL penicillin and 0.1 μg/mL streptomycin. Every 72 h thereafter, the medium was replaced with DMEM supplemented with 1 μg/mL of insulin until adipocyte differentiation was fully induced (day 8). Complete differentiation of 3T3-L1 cells was confirmed by the presence of a fat droplet occupying approximately 80–90% of the adipocytes, at which point the cells were considered mature adipocytes.

### 2.4 Evaluation of Cell Viability and TXA Effects on Adipocyte Glucose Uptake

The viability of 3T3-L1 adipocyte was assessed using the MTT assay, as previously described.^29^ Cells were seeded in 96-well plates at a density of 1 × 10^5^ cells per well and incubated with DMEM containing 0.5–200 μM TXA or its vehicle for 48 h. TXA was dissolved in DMSO and diluted in PBS prior to cell treatment. The final concentration of DMSO was 0.01% (v/v) and was matched across all experimental groups, including vehicle controls. After incubation, a 0.5 mg/mL MTT solution in phosphate-buffered saline (PBS) was added and incubated for 2 h at 37 °C. To determine cell viability, the medium was replaced with 100 μL DMSO for full solubilizing of the formazan crystals, and absorbance was measured at 570 nm using a microplate reader (Asys UVM340, Biochrom, Cambridge, UK). TXA was dissolved in DMSO, with the final DMSO concentration maintained at ≤0.2%. Each experiment was performed in triplicate.

To evaluate the TXA effect on adipocyte glucose uptake, 3T3-L1 adipocytes were seeded at a density of 1 × 10^5^ cells/well, and incubated in KRPH buffer (pH 7.4) for 4 h. The cells were then treated with 12.5, 25, and 50 TXA μM TXA, or 20 μM rosiglitazone (RGZ) for 24 h. Afterward, the culture medium was discarded and replaced with 900 μL of KRPH, with or without 100 nM insulin, for 30 min at 37 °C. Subsequently, 100 μM 2-NBDG was added and incubated for 30 min. The cells were lysed in 0.1 M KPB buffer (pH 10) containing 0.1% Triton X-100.^30^ The fluorescence intensity of 2-NBDG-labeled cells was analyzed using a fluorescence microplate reader (BioTek Instruments, Winooski, VT, USA) with excitation at 485 nm and emission at 535 nm.

### 2.5 TNF-α-Induced Insulin Resistance (IR) in 3T3-L1 Adipocytes and Assessment of Glucose Uptake

3T3-L1 adipocytes were seeded at a density of 1 × 10^5^ cells/well and incubated in KRPH buffer (pH 7.4) for 4 h. The cells were then treated with 10 ng/mL TNF-α for 24 h.^31^ TNF-α stock solution (100 μg/mL) was prepared according to the manufacturer’s instructions and diluted in culture medium to achieve a final concentration of 10 ng/mL. Afterward, the culture medium was discarded and replaced with 900 μL of KRPH, with or without 100 nM insulin, for 30 min at 37 °C. 2-NBDG was used to evaluate glucose uptake as described before.^30^ The induction of IR was confirmed by assessing insulin-stimulated glucose uptake.

To evaluate the effect of TXA on TNF-α-induced reductions in glucose uptake, cells were treated with vehicle, 12.5, 25, and 50 μM TXA, or 20 μM RGZ for 24 h, followed by cotreatment with 10 ng/mL TNF-α for an additional 24 h.

### 2.6 Western Blot Analysis

3T3-L1 adipocytes were treated as described in Section 2.5, with pretreatment with TXA (50 μM) or rosiglitazone (20 μM) for 24 h, followed by cotreatment with TNF-α (10 ng/mL) for an additional 24 h. The same treatment scheme was applied in all subsequent assays to ensure consistency and comparability across experiments. The total cellular protein from adipocytes cotreated with 50 μM TXA or 20 μM RGZ and 10 ng/mL TNF-α was isolated using RIPA lysis buffer containing 1% protease inhibitor cocktail. The homogenate was centrifuged at 12,000*g* for 15 min at 4 °C, and the supernatant was stored at −80 °C.

To prepare the plasm membrane fractions for GLUT4 quantification, the cells were washed with cold PBS, scraped off the plates into homogenization buffer (Tris-HCl 10 mM; EDTA 1 mM; sucrose 250 mM, pH 7.4) and centrifugated at 1000*g* for 10 min at 4 °C. The supernatant was ultracentrifuged at 150,000*g* for 75 min at 4 °C and the pellet was suspended with homogenization buffer and kept at −80 °C.^32^

Total protein (20 μg) was separated on 8% SDS-PAGE and transferred onto a PVDF membrane (Millipore, USA). Incubation with primary antibodies (1:1000) pIRS1, IRS1, PI3K, pAkt, Akt, GLUT4, pAMPKα, AMPKα, pJNK, JNK, pNF-κB, NF-κB and β-actin was at 4 °C overnight, followed by incubation with HRP-linked secondary antibodies (1:3000) at room temperature for 2 h. Amersham ECL Prime Western Blotting Detection Reagent (Bio-Rad Laboratories, USA) was used. ChemiDoc Image System with Image Lab 5.1 software (Bio-Rad Laboratories, USA) was used to acquire and analyze Western blot images. Arbitrary optical density units of the targeting protein were normalized against the control, the average value of the control was set at 1, and the results were expressed as fold change of the control.

### 2.7 Oxidative Stress Assays

Intracellular reactive oxygen species (ROS) levels were assessed using the DCFH-DA fluorescent probe. The cells were washed with KRP buffer (pH 7.4) and incubated with 5 μM DCFH-DA and 5 μM glucose for 30 min. Fluorescence intensity was measured immediately using a fluorescence microplate reader (Asys UVM340, Biochrom, UK), with excitation at 480 nm and emission at 525 nm.^33^

To evaluate antioxidant effects, cells were harvested and centrifuged to obtain cell pellets. The pellets were resuspended in three volumes of protein extraction buffer containing a protease inhibitor cocktail, followed by sonication. The homogenates were then centrifuged at 12,000*g* for 10 min at 4 °C, and the resulting supernatants were used for subsequent biochemical analyses.

Lipid peroxidation was assessed by measuring malondialdehyde (MDA) levels according to the method described by Uchiyama and Mihara,^34^ based on the formation of a colored adduct resulting from the reaction between thiobarbituric acid and MDA, which was quantified spectrophotometrically at 535 nm. Reduced glutathione (GSH) content was determined using the method described by Ellman,^35^ which relies on the reaction between sulfhydryl groups and 5,5′-dithiobis­(2-nitrobenzoic acid), producing a yellow-colored compound measured at 412 nm. Catalase (CAT) activity was measured by monitoring the decrease in absorbance at 240 nm due to the decomposition of hydrogen peroxide (H_2_O_2_).^36^ Superoxide dismutase (SOD) activity was evaluated using the method described by Marklund,^37^ based on the ability of SOD to inhibit pyrogallol autoxidation. Absorbance was recorded at 420 nm at 1 min intervals for 3 min. Nitrite levels, used as an indicator of nitric oxide production, were determined using the Griess reaction,^38^ with absorbance measured at 540 nm. Nitrite concentrations were calculated from a sodium nitrite standard curve. Protein concentrations were determined using the Lowry method.^39^ All measurements were normalized to total protein content and expressed relative to the control group.

### 2.8 Leptin and Adiponectin Determination

The levels of leptin (#EZML-82K) and adiponectin (#EZMADP-60K) secreted into the cell culture medium were measured using ELISA kits (Merck Millipore, USA) according to the manufacturer’s protocol.

### 2.9 Assessment of Cellular Lipid Content

Intracellular lipid accumulation in 3T3-L1 adipocytes was measured using the Oil Red O assay.^40^ The cells were washed with PBS and fixed with 4% formaldehyde in PBS for 1 h, then stained with Oil Red O solution (60% isopropanol and 40% water) for 2 h and thoroughly rinsed with distilled water. The retained dye was extracted using 60% isopropanol, and absorbance was measured at 510 nm using a microplate reader.

### 2.10 Determination of Lipolysis

Lipolysis was evaluated by measuring the amount of glycerol released into the culture media.^41^ After treatment, the cell medium was removed, and glycerol was quantified using a combination of the Malaprade reaction and the Hantzsch reaction. This involved the use of 1.8% sodium periodate and 7.7% ammonium acetate in acetic acid, as well as 1% acetylacetone in isopropyl alcohol. The absorbance was read at 410 nm, and the glycerol concentration was calculated based on a glycerol standard curve.

### 2.11 Quantitative Real-Time Polymerase Chain Reaction (qRT-PCR)

Total RNA was isolated using QIAzol lysis reagent, according to the manufacturer’s protocol and the quality and quantity of the RNA were determined spectrophotometrically at 260 and 280 nm (Nanodrop 2000, Thermo Fisher Scientific, USA). The total RNA (2 μg) was used for the cDNA synthesis. A High-Capacity cDNA Reverse Transcription kit (Thermo Fisher Scientific, USA) was used for the reverse-transcribe RNA to cDNA according to the manufacturer’s protocol. Real-time PCR was performed with an Mx3005p PCR system (Agilent, USA) using GoTaq master mix kit with SYBER green (Promega, USA) according to the protocol provided by the manufacturer. PCR reactions consisted of an initial denaturation cycle at 95 °C for 10 min, followed by 40 amplification cycles: 15 s at 95 °C and 1 min at 60 °C. The primers used are shown in Table 1. Relative mRNA levels of target genes were normalized to the β-actin gene by employing the comparative threshold cycle (2^–ΔΔ^CT) method.

**Table 1:** Primer Sequences Used for the qRT-PCR Analysis^a^

gene	forward primer (5′-3′) reverse primer (3′-5′)
PPARγ	GCTGAACGTGAAGCCCATCG
	TTCTGGAGCACCTTGGCGAA
PLIN1	GCACCATCTCTACCCGCCTT
	CGATGCTTCCCAGAGCCAGA
ATGL	TTGTTGGAGTGGCTGCCTGA
	GCTGACGCTGGCATTCTTCC
HSL	GCCTGGCAAAATCTGAGGGC
	TCACAGTGCTTGACAGCCCA
ACSL1	CCATGAGCTGTTCCGGTATTT
	CCGAAGCCCATAAGCGTGTT
CPT1b	ATGTATCGCCGCAAACTGGACC
	CTCTGAGAGGTGCTGTAGCAAG
ACOX1	GCCATTCGATACAGTGCTGTGAG
	CCGAGAAAGTGGAAGGCATAGG
ACADM	AGGATGACGGAGCAGCCAATGA
	GCCGTTGATAACATACTCGTCAC
PPARGC1A	CTCTCCTTGCAGCACCAGAA
	CAATGAATAGGGCTGCGTGC
NRF1	GATATCGGACAGCGCAGTCA
	GCGTTTCTCACTCCACCAGA
TFAM	GCTTCCAGGAGGCAAAGGAT
	TGCTCAGAGATGTCTCCGGA
β-actin	ACCCCAGCCATGTACGTAGC
	AGCTGTGGTGGTGAAGCTGT

a ATGL, adipose triglyceride lipase; HSL, hormone-sensitive lipase; ACSL1, acyl-CoA synthetase long-chain family member 1; CPT1b, carnitine palmitoyltransferase 1b; ACOX1, acyl-coenzyme A oxidase 1; ACADM, acyl-coenzyme A dehydrogenase; PPARGC1A, peroxisome proliferative activated receptor γ, coactivator 1 α; NRF1, nuclear respiratory factor-1; TFAM, transcription factor A mitochondrial.

### 2.12 Statistical Analysis

All experiments were carried out in triplicate. Data are reported as mean ± standard deviation (SD). Statistical analysis was performed with GraphPad Prism 8.0 software (San Diego, USA). One-way analysis of variance (ANOVA) followed by the Tukey’s test was performed to compare multiple groups. *P* < 0.05 was considered statistically significant.

## 3 Results

### 3.1 TXA Enhances Glucose Uptake in Noninsulin-Resistant and TNF-α Insulin-Resistant (IR) Adipocytes without Inducing Cytotoxicity

The cytotoxicity of TXA in 3T3-L1 adipocytes was evaluated using the MTT assay. As shown in Figure 1B, TXA at concentrations ranging from 0.5–200 μM did not significantly affect cell viability following 48 h of treatment. Based on this result and prior reports on the efficacy of other pentacyclic triterpenes in 3T3-L1 cells,^27,42^ concentrations of 12.5, 25, and 50 μM were selected for subsequent experiments.

To examine the effect of TXA on glucose uptake under both physiological and insulin-resistant conditions, a 2-NBDG fluorescent glucose analog assay was conducted in mature adipocytes. Under physiological conditions, treatment with insulin, TXA (50 μM), or RGZ (20 μM) each significantly increased glucose uptake by 57% and 47% compared to untreated control cells (Figure 1C).

In TNF-α-induced IR adipocytes, exposure to TNF-α (10 ng/mL) reduced insulin-stimulated glucose uptake by 40% compared to the insulin-treated group. Treatment with TXA at 12.5, 25, and 50 μM significantly restored glucose uptake by 27%, 34%, and 35%, respectively, relative to the TNF-α + insulin group (Figure 2). Importantly, TXA treatment also significantly increased glucose uptake when compared directly to the TNF-α–only group, reaching levels comparable to those observed in the TNF-α + insulin condition (Figure 2).

**Figure 2:**
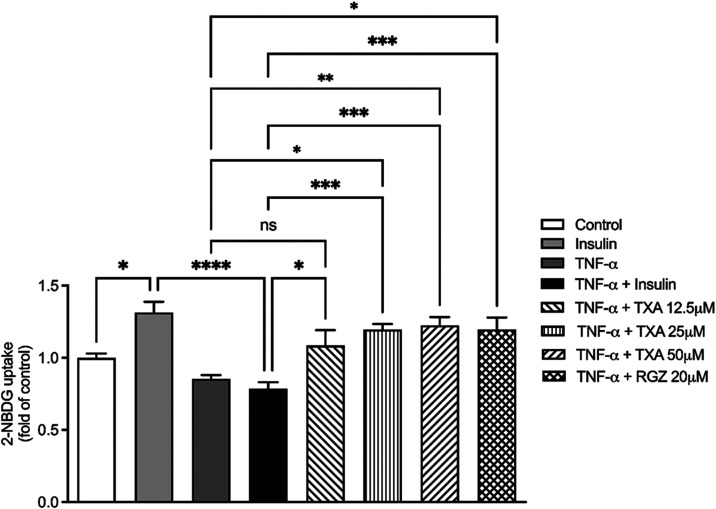
Taraxasterol acetate (TXA) restored glucose uptake in TNF-α-induced insulin-resistant (IR) adipocytes. Glucose uptake was measured using the 2-NBDG assay. The data are expressed as the mean ± SD (*n* = 3). *p < 0.05, **p < 0.01, ****p < 0.0001 (ANOVA followed by Tukey test).

The positive control, rosiglitazone (RGZ, 20 μM), similarly restored glucose uptake both relative to the TNF-α + insulin group and when compared directly to TNF-α alone (Figure 2).

### 3.2 TXA Enhances Glucose Uptake through Activation of Insulin Signaling and AMPK in TNF-α IR Adipocytes

To elucidate the mechanisms underlying TXA-mediated glucose uptake in TNF-α IR adipocytes, we evaluated its effects on key components of the insulin signaling pathway, including IRS1, PI3K, AKT and the membrane protein expression of GLUT4. Additionally, we assessed its impact on AMPK expression.

As shown in Figure 3A, insulin treatment (100 nM) significantly decreased IRS1 (Ser 307) phosphorylation (by 3.33-fold), while increasing phosphorylation of PI3K and pAKT by 2.13-fold and 11.5-fold, respectively. This led to enhanced GLUT4 translocation to the plasma membrane compared to the control group. TNF-α disrupted insulin signaling by increasing IRS1 (Ser 307) phosphorylation (by 8.0-fold) and decreasing phosphorylation of PI3K (by 3-fold) and p AKT (by 10.5-fold), resulting in a 50% decrease in GLUT4 translocation compared to the insulin-treated group.

**Figure 3:**
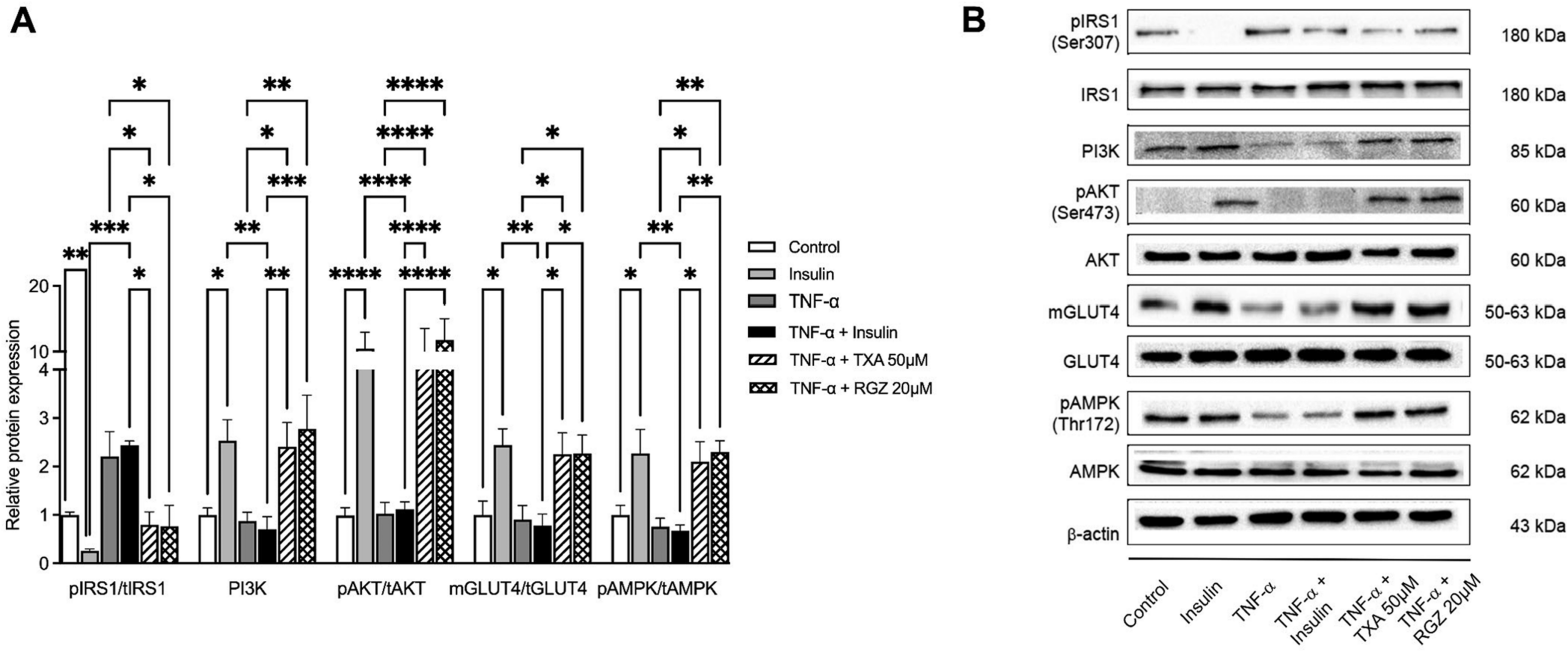
Taraxasterol acetate (TXA) improved the protein expression related to insulin signaling and AMPK in TNF-α-induced IR adipocytes. (A) The protein expression levels of IRS1, PI3K, AKT, GLUT4, and AMPK were assessed by Western blotting analysis. (B) Densitometry analysis of the Western blots showing relative protein expression normalized to β-actin and expressed as fold change relative to the control group (Figures S6–S8, Supporting Information). β-Actin was used as a loading control. Data are presented as the mean ± SD (*n* = 3). *p < 0.05, **p < 0.01, ***p < 0.001, ****p < 0.0001 (ANOVA followed by Tukey test).

Treatment with TXA (50 μM) and RGZ (20 μM) significantly reversed these effects. TXA reduced IRS1 (Ser 307) phosphorylation by 2.5-fold and increased phosphorylation of PI3K and AKT by 2.7-fold and 10-fold, respectively. This restoration promoted GLUT4 translocation by 1.9-fold compared to the TNF-α + insulin group (Figure 3A). Importantly, comparable rescue effects were observed when TXA- and RGZ-treated cells were directly compared with the TNF-α–only condition. Similar improvements were observed with RGZ. Representative immunoblots illustrating these signaling changes are shown in Figure 3B.

In parallel, insulin treatment enhanced AMPK phosphorylation by 1.36-fold relative to the control group, whereas TNF-α suppressed this activation by 2.0-fold. Both TXA and RGZ restored AMPK phosphorylation to levels comparable to the insulin-only group, increasing phosphorylation by 1.9-fold compared to the TNF-α treated adipocytes (Figure 3A,B).

### 3.3 TXA Inhibits TNF-α-Mediated Inflammatory Protein Expression in IR Adipocytes

To assess the anti-inflammatory effects of TXA, we analyzed the phosphorylation of JNK and NF-κB, key mediators of TNF-α-induced inflammation (Figure 4A). TNF-α significantly increased the phosphorylation of JNK and NF-κB by 2.2-fold and 2.4-fold, respectively, relative to the control group.

**Figure 4:**
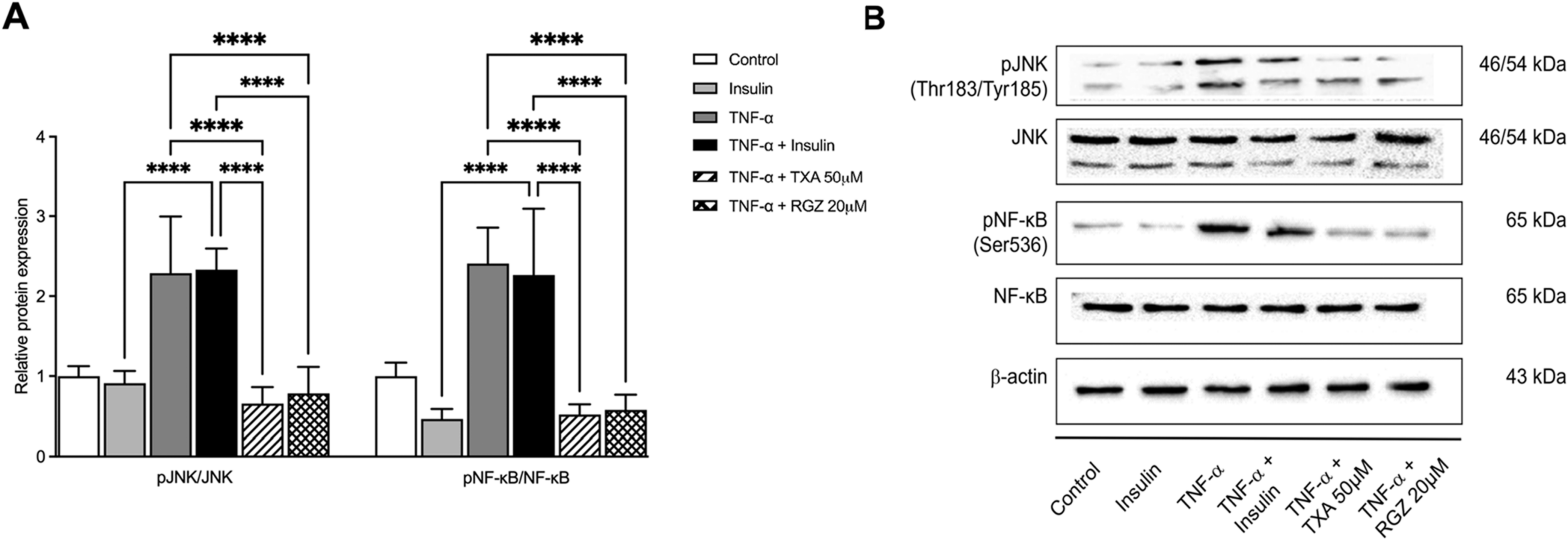
Taraxasterol acetate (TXA) attenuated pro-inflammatory protein expression induced by TNF-α in adipocytes. (A) The protein expression levels of JNK and NF-κB were assessed by Western blotting analysis. (B) Densitometry analysis of the Western blots showing relative protein expression normalized to β-actin and expressed as fold change relative to the control group (Figure S9, Supporting Information). β-Actin was used as a loading control. Data are presented as the mean ± SD (*n* = 3). ****p < 0.0001 (ANOVA followed by Tukey test).

TXA (50 μM) markedly inhibited these effects, reducing JNK phosphorylation by 3.3-fold and NF-κB phosphorylation by 4.6-fold compared with the TNF-α + insulin group (Figure 4A). These inhibitory effects were also significant when TXA-treated cells were directly compared with the TNF-α–only condition. RGZ treatment produced similar reductions (2.9-fold for JNK and 4.2-fold for NF-κB), indicating the anti-inflammatory potential of TXA. Comparable results were observed when RGZ-treated cells were compared with TNF-α alone. Representative immunoblots illustrating these changes are shown in Figure 4B.

### 3.4 TXA Attenuates Oxidative Stress and Reduces ROS Levels in IR Adipocytes

The effect of TXA on oxidative stress was evaluated by measuring intracellular levels of reactive oxygen species (ROS) and antioxidant defense markers. TNF-α increased ROS production, as indicated by elevated DCF-DA fluorescence, malondialdehyde (MDA), and nitrite levels. It also decreased glutathione (GSH) levels and the enzymatic activities of superoxide dismutase (SOD) and catalase (CAT), compared to the control group (Table 2).

**Table 2:** Effects of Taraxasterol Acetate (TXA) on Markers of Oxidative Stress in TNF-α-Treated Adipocytes^a^

parameter	control	TNF-α	TNF-α + TXA 12.5 μM	TNF-α + TXA 25 μM	TNF-α + TXA 50 μM	TNF-α + RGZ 20 μM
DCF-DA (fluorescence intensity)	1.00 ± 0.27	1.56 ± 0.30[Table-fn t2fn3]	1.41 ± 0.44	1.08 ± 0.22[Table-fn t2fn5]	1.06 ± 0.22[Table-fn t2fn5]	1.17 ± 0.30[Table-fn t2fn6]
MDA (μM/μg protein)	0.73 ± 0.24	1.67 ± 0.22[Table-fn t2fn4]	1.60 ± 0.41	1.14 ± 0.51[Table-fn t2fn5]	0.43 ± 0.14[Table-fn t2fn6]	0.57 ± 0.32[Table-fn t2fn7]
GSH (μM/μg protein)	1.00 ± 0.08	0.51 ± 0.12[Table-fn t2fn2]	0.42 ± 0.14	0.53 ± 0.38	0.89 ± 0.09[Table-fn t2fn6]	0.95 ± 0.20[Table-fn t2fn6]
SOD (U/μg protein)	4.04 ± 0.86	1.55 ± 0.26[Table-fn t2fn3]	2.40 ± 0.18	2.46 ± 0.26	4.35 ± 1.14[Table-fn t2fn5]	4.31 ± 1.30[Table-fn t2fn6]
CAT (mmol/min/μg protein)	11.8 ± 1.37	0.90 ± 0.47[Table-fn t2fn3]	1.26 ± 0.31	3.14 ± 0.89	11.66 ± 4.27[Table-fn t2fn5]	13.41 ± 6.63[Table-fn t2fn6]
nitrite (μM/μg protein)	4.44 ± 1.57	11.25 ± 0.69[Table-fn t2fn3]	8.92 ± 1.17	7.88 ± 4.72	3.24 ± 1.82[Table-fn t2fn5]	4.97 ± 2.21[Table-fn t2fn6]

a Data are reported as means ± SD (*n* = 3).

* *p* < 0.05.

** *p* < 0.01.

*** *p* < 0.001 significant differences versus control group.

# *p* < 0.05.

## *p* < 0.01.

### *p* < 0.001 significant differences versus TNF-α group.

TXA (50 μM) and RGZ (20 μM) significantly reversed these alterations, restoring DCF-DA, MDA, and nitrite levels, as well as GSH content and SOD and CAT activity. At 25 μM, TXA exhibited partial antioxidant effects by reducing only DCF-DA and MDA levels. These results suggest that TXA enhances the oxidative stress response in insulin-resistant adipocytes.

### 3.5 TXA Improves Leptin and Adiponectin Levels in TNF-α IR Adipocytes

We investigated the influence of TXA on adipokine production by quantifying intracellular leptin and adiponectin levels. TNF-α treatment resulted in a 101% increase in leptin and a 75% reduction in adiponectin levels compared to the control group.

TXA treatment at 12.5, 25, and 50 μM reduced leptin levels by 18%, 40%, and 44%, respectively, relative to the TNF-α group (Figure 5A). Additionally, adiponectin levels were significantly restored by TXA at 25 and 50 μM, with increases of 120%, and 351%, respectively (Figure 5B). RGZ (20 μM) also reverses TNF-α-induced adipokine imbalance, increasing adiponectin by 390% and reducing leptin by 44%.

**Figure 5:**
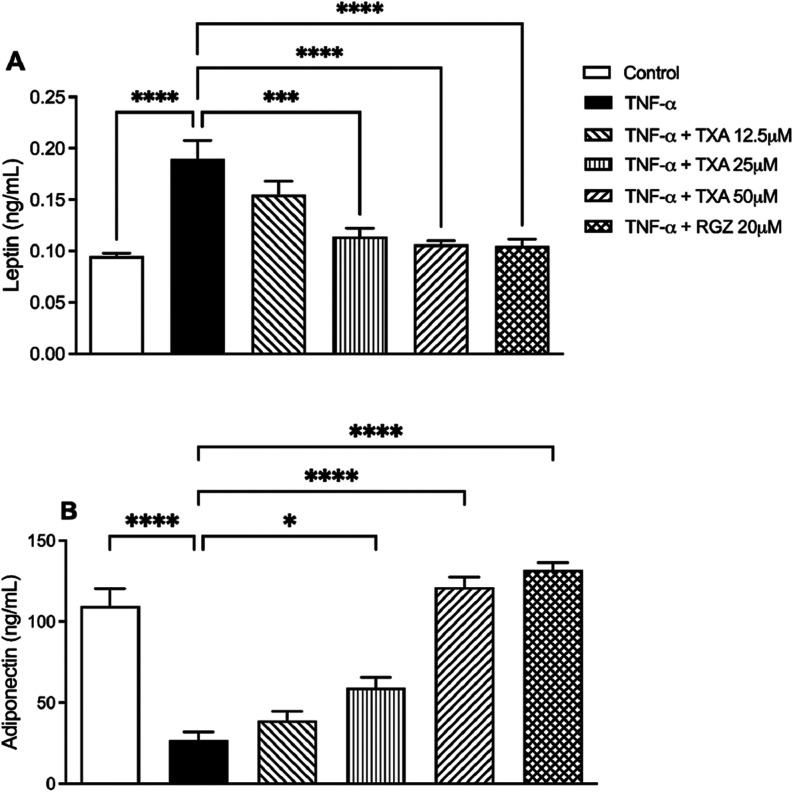
Taraxasterol acetate (TXA) restored the leptin and adiponectin levels in TNF-α-induced IR adipocytes. (A) Leptin and (B) Adiponectin levels were measured by ELISA. Data are presented as the mean ± SD (*n* = 3). *p < 0.05, ***p < 0.001, ****p < 0.0001 (ANOVA followed by Tukey test).

### 3.6 TXA Inhibits Lipolysis in TNF-α IR Adipocytes

To examine the effect of TXA on lipid metabolism, we assessed intracellular lipid accumulation using Oil Red O staining. TNF-α decreased cellular lipid content by 26% compared to control cells, as evidenced by reduced lipid droplet density in stained photomicrographs (Figure 6). Treatment with TXA at 12.5, 25, and 50 μM or RGZ (20 μM) prevented this lipid loss and preserved lipid droplet density (Figure 6).

**Figure 6:**
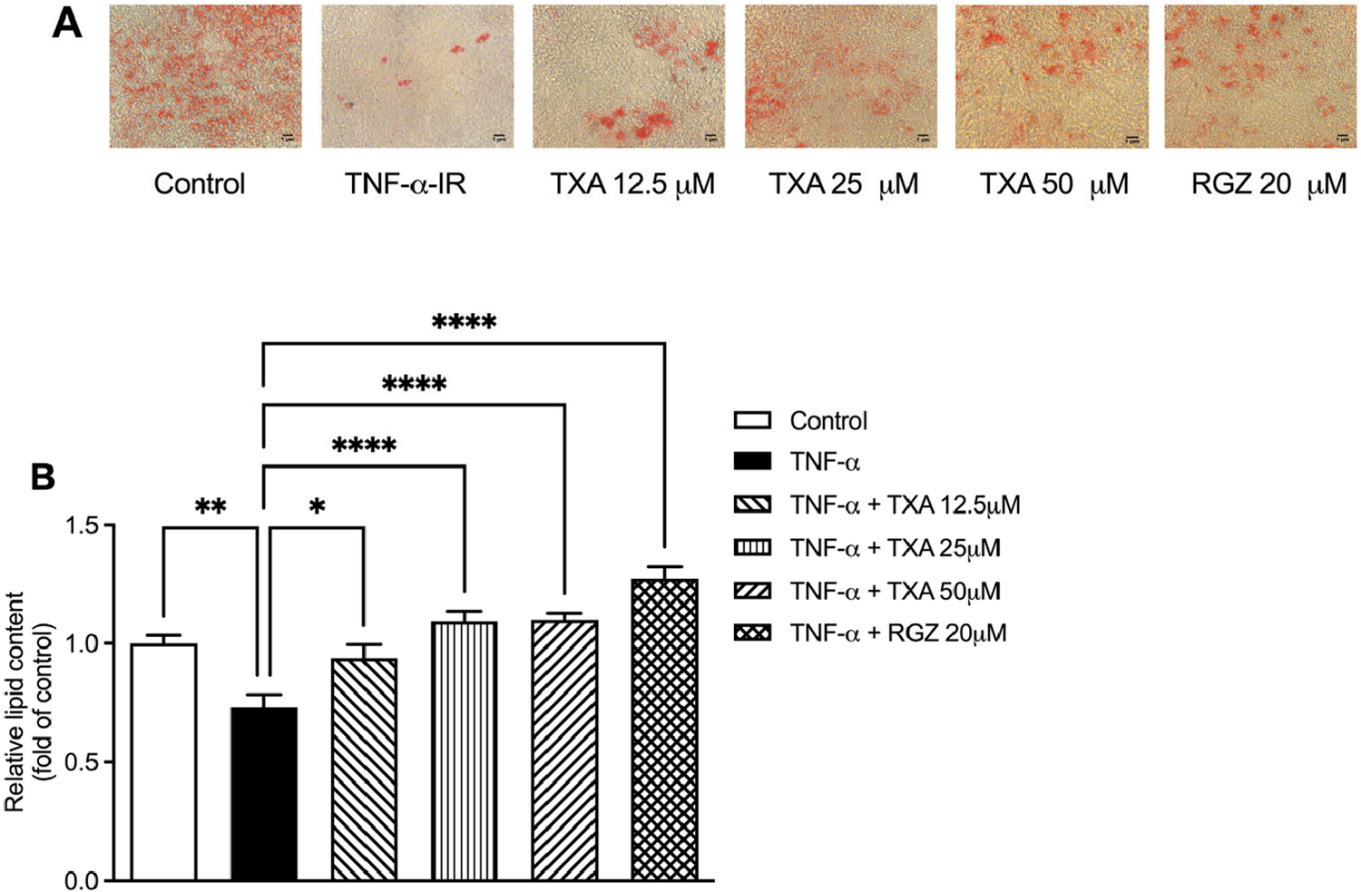
Taraxasterol acetate (TXA) modulated lipid accumulation by reversing TNF-α-induced lipolytic activity in IR adipocytes. Intracellular lipid accumulation was evaluated by Oil Red O staining. (A) Microphotographs of Oil Red O staining. (B) Relative lipid content was determined by spectrophotometric quantification of Oil Red O staining. Data are presented as the mean ± SD (*n* = 3). *p < 0.05, **p < 0.01, ****p < 0.0001 (ANOVA followed by Tukey test).

Furthermore, TNF-α significantly increased lipolysis, as indicated by a 340% rise in glycerol release into the culture medium (Figure 7A). TXA at 25 and 50 μM reduced glycerol release by 61.9% and 85.6%, respectively. RGZ (20 μM) also inhibited lipolysis, decreasing glycerol release by 80%.

**Figure 7:**
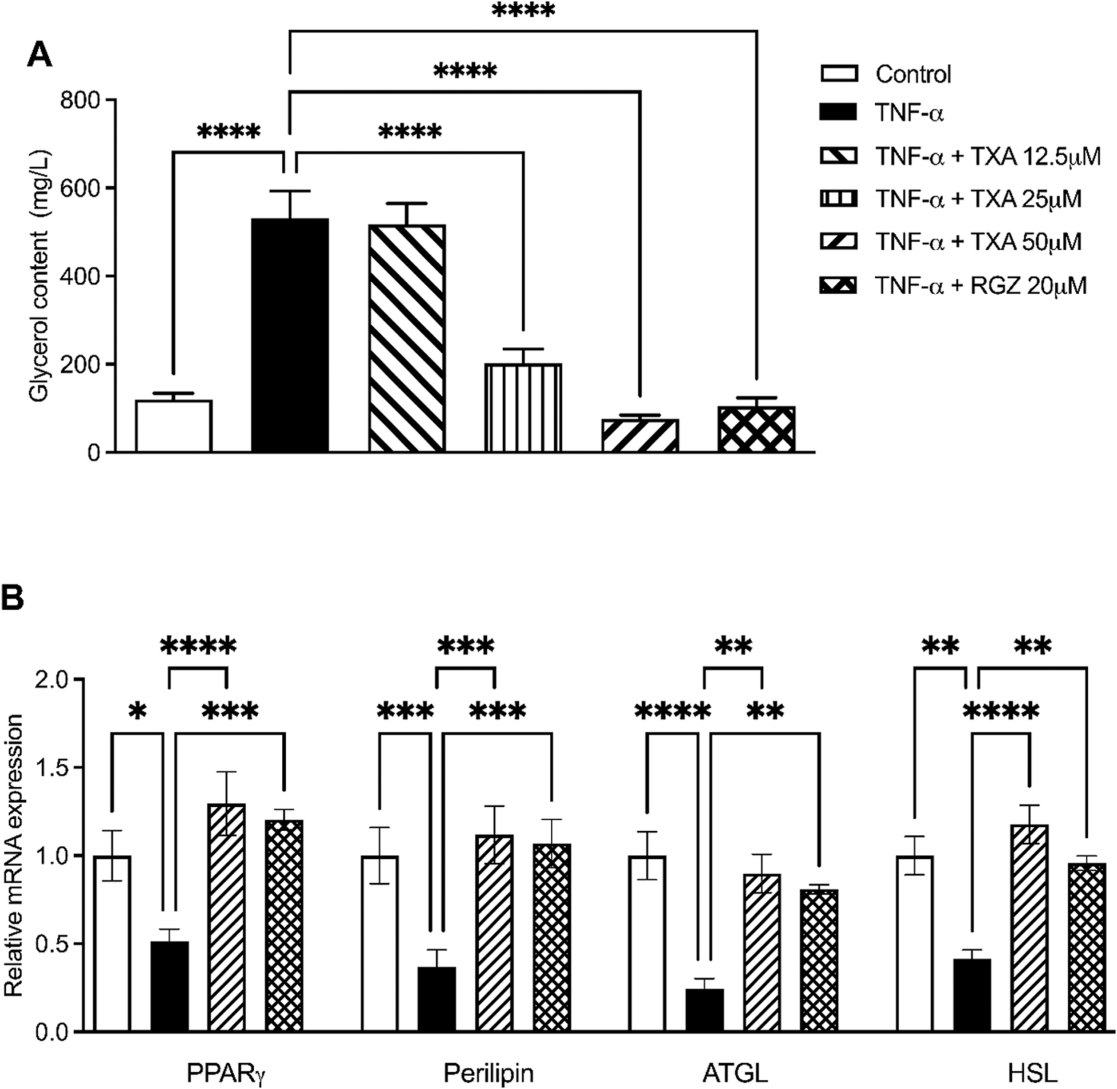
Taraxasterol acetate (TXA) attenuated TNF-α–induced lipolysis by regulating key lipolytic genes in IR adipocytes. (A) Glycerol content. The glycerol content was quantified by Malaprade-Hantzsch reactions. (B) Relative mRNA expression levels of PPARγ, PLIN1, ATGL, and HSL. Gene expression was analyzed by quantitative real-time PCR (qRT-PCR). Data are presented as the mean ± SD (*n* = 3). *p < 0.05, **p < 0.01, ***p < 0.001, ****p < 0.0001 (ANOVA followed by Tukey test).

We next evaluated gene expression related to lipolysis. TNF-suppressed the mRNA expression of PPARγ, HSL, ATGL, and perilipin by 2.5-fold, 2.7-fold, 5.50-fold and 2.5-fold, repectively. TXA (50 μM) restored the expression of these genes, increasing PPARγ by 2.5-fold, perilipin by 3.1-fold, ATGL by 4.6-fold, and HSL by 2.8-fold compared to the TNF-α group (Figure 7B). RGZ (20 μM) exerted comparable effects.

### 3.7 TXA Promotes Mitochondrial Biogenesis and Fatty Acid β-Oxidation in TNF-α IR Adipocytes

To determine the impact of TXA on mitochondrial function, we measured mRNA expression of key genes involved in fatty acid β-oxidation (ACSL1, CPT1b, ACOX1, and ACADM) and mitochondrial biogenesis (PPARGC1A, NRF1, and TFAM). TNF-α significantly downregulated the expression of all these gene compared to the control. TXA (50 μM) restored the expression of all genes except NRF1. In contrast, RGZ (20 μM) fully reversed TNF-α-induced suppression across all measured targets (Figure 8). These results suggest that TXA supports mitochondrial health and enhances lipid metabolism under inflammatory conditions.

**Figure 8:**
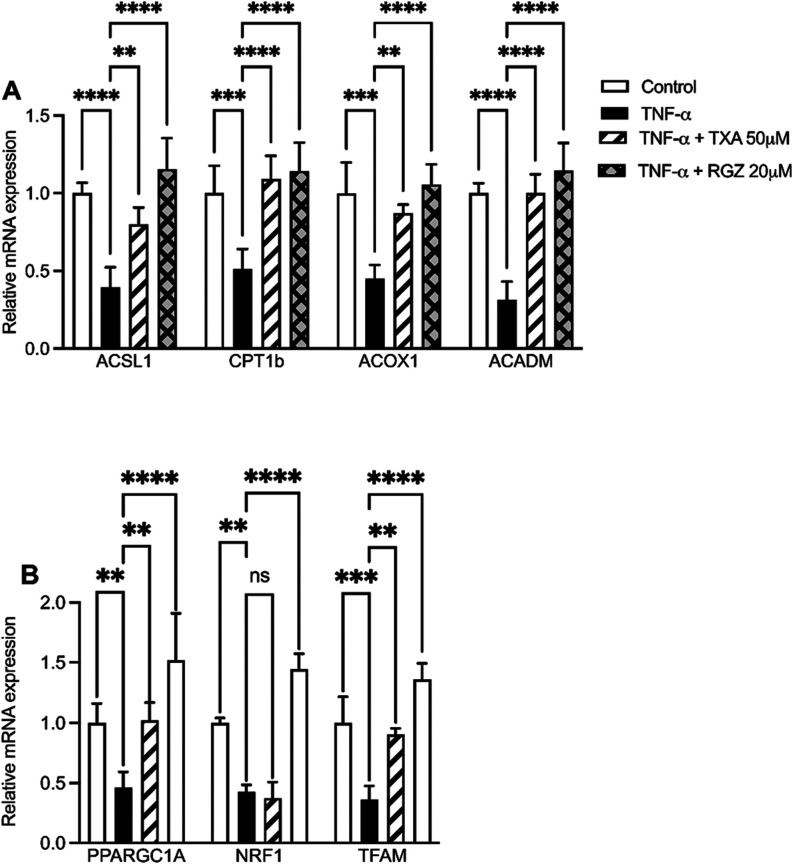
Taraxasterol acetate (TXA) prevented TNF-α–induced downregulation of β-oxidation and mitochondrial biogenesis-related genes in IR adipocytes. Gene expression was analyzed by quantitative real-time PCR (qRT-PCR). (A) Relative mRNA expression levels of ACSL1, CPT1b, ACOX1, and ACADM. (B) Relative mRNA expression levels of PGC1, NRF1, and TFAM. Data are presented as the mean ± SD (*n* = 3). **p < 0.01, ***p < 0.001, ****p < 0.0001 (ANOVA followed by Tukey test).

## 4 Discussion

Insulin resistance (IR) is a central feature of metabolic disorders such as obesity and type 2 diabetes mellitus (T2DM), arising primarily from impaired insulin signaling in key metabolic tissues.^1,2^ Chronic low-grade inflammation in adipose tissue has been widely recognized as a major contributor to IR, as first demonstrated by the identification of elevated TNF-α levels in obese rodents and the subsequent improvement in insulin sensitivity upon TNF-α inhibition.^5,43,44^ Current therapeutic approaches, including lifestyle modifications and insulin sensitizers such as thiazolidinediones, have demonstrated clinical benefits but are limited by adverse effects, underscoring the need for safer alternatives that target both metabolic and inflammatory components of IR.

Pentacyclic triterpenes, such as ursolic acid, oleanolic acid, and lupeol, exhibit anti-inflammatory, antioxidant, and insulin-sensitizing properties, improving glucose homeostasis by enhancing insulin secretion and signaling, promoting GLUT4-mediated glucose uptake, and activating pathways including IRS-1/PI3K/Akt and AMPK.^16^ Consistent with this pharmacological profile, the present study demonstrates that taraxasterol acetate (TXA), a pentacyclic triterpene isolated from *E. ballotaefolium*, effectively attenuates TNF-α-induced insulin resistance and lipolysis in 3T3-L1 adipocytes.

TNF-α is a key cytokine elevated in obesity that disrupts adipocyte metabolic function by impairing insulin receptor signaling, reducing GLUT4 expression, stimulating lipolysis, and promoting chronic inflammation.^43,44^ In line with previous studies, TNF-α in our model induced serine phosphorylation of IRS-1 and inhibited PI3K expression and Akt phosphorylation, ultimately impairing GLUT4 translocation and reducing glucose uptake. TXA significantly reversed these alterations by reducing IRS-1 serine phosphorylation and restoring PI3K/Akt activation and GLUT4 translocation. These effects resemble those described for structurally related triterpenes such as ursolic and asiatic acids, which enhance glucose uptake and restore insulin signaling via PI3K/Akt pathway modulation.^45,46^ Rosiglitazone (RGZ), used as a positive control, elicited similar improvements in insulin signaling,^47,48^ confirming TXA’s comparable efficacy. Direct statistical comparisons between TXA (50 μM) and RGZ revealed no significant differences across the evaluated metabolic and signaling parameters.

Inflammatory signaling pathways, including JNK, ERK, and NF-κB, play a crucial role in TNF-α-induced insulin resistance.^7–9^ In this study, TXA significantly inhibited TNF-α-induced activation of JNK and NF-κB, thereby preventing IRS-1 serine phosphorylation and restoring downstream insulin signaling. This anti-inflammatory effect, shared by RGZ and other triterpenes,^49,50^ highlights TXA’s dual insulin-sensitizing and inflammation-modulating potential.

Oxidative stress is another factor that amplifies inflammation and insulin resistance. TNF-α increased ROS, MDA, and nitrite levels in adipocytes while decreasing GSH content and antioxidant enzyme activity (SOD and CAT), contributing to metabolic dysfunction.^51,52^ TXA mitigated these effects, restoring redox balance to levels comparable to untreated controls. These data are consistent with reports showing that TXA and other triterpenes exhibit robust antioxidant activity and inhibit ROS-generating inflammatory pathways, including NF-κB and JNK.^53,54^

Adipokines such as adiponectin and leptin regulate metabolic homeostasis.^55,56^ TNF-α suppressed adiponectin and elevated leptin, promoting lipolysis and inhibiting lipogenesis, changes associated with inflammation and IR.^57,58^ TXA restores adiponectin and normalized leptin levels, likely via PPAPγ activation and JNK inhibition, a mechanism also reported for other triterpenes and PPARγ agonists.^59,60^ TNF-α disrupted lipid metabolism by stimulating lipolysis and suppressing genes like PPARγ, perilipin, ATGL, and HSL, reducing lipid content and increasing glycerol release.^61–63^ TXA countered these effects, preserving lipid storage and gene expression. This mirrors the action of RGZ, which promotes lipid accumulation and insulin sensitivity through PPARγ signaling.^64^ Anti-inflammatory actions and direct transcriptional regulation of lipolytic genes likely mediate TXA’s antilipolytic effects. Although TXA increased PPARγ expression and lipid accumulation in adipocytes, the present findings do not support its classification as a classical thiazolidinedione-type PPARγ agonist. Notably, this study was not designed to evaluate systemic or long-term effects. Accordingly, potential adverse effects commonly associated with thiazolidinediones cannot be excluded and warrant further investigation in appropriate in vivo and mechanistic studies. Nevertheless, the ability of TXA to improve insulin signaling and metabolic homeostasis in adipocytes suggests its potential relevance as a modulator of insulin resistance.

TXA modulated the expression of genes associated with fatty acid β-oxidation and mitochondrial biogenesis, processes essential for the maintenance of adipocyte metabolic function. TNF-α suppressed the expression of key genes involved in β-oxidation (Acsl1, Cpt1b, Acox1, and Acadm) and mitochondrial biogenesis and function (Ppargc1a, Nrf1, and Tfam), whereas TXA restored their expression. These transcriptional changes are consistent with pathways associated with enhanced mitochondrial capacity and lipid metabolism and are in agreement with previous studies reporting that triterpenes regulate metabolic gene programs through PPAR-α– and PGC-1α–related signaling mechanisms.^65–67^

Finally, TXA activated AMPK, a master regulator of cellular energy balance. AMPK promotes glucose uptake, inhibits lipogenesis and lipolysis, and supports mitochondrial biogenesis while suppressing inflammation.^68,69^ TXA’s metabolic and anti-inflammatory effects may therefore by partly mediated by AMPK activation, a mechanism shared with RGZ and other triterpenes.^16,70^

In conclusion, the present results indicate that taraxasterol acetate (TXA) alleviates TNF-α-induced insulin resistance in adipocytes through multiple mechanisms: it enhances insulin signaling, reduces inflammation and oxidative stress pathways, normalizes adipokine secretion, improves lipid metabolism, promotes mitochondrial biogenesis, and activates AMPK. These coordinated actions support improved glucose uptake, reduced lipolysis, and enhanced energy homeostasis. Collectively, these findings underscore the potential of TXA as a modulator of adipocyte function and lipid homeostasis, with promising implications for the management of insulin resistance and related metabolic disorders.

## Supplementary Material



## Notes

FT-IR, MS (70 eV), ^1^H NMR (500 MHz, CDCl_3_), ^13^C NMR (125 MHz, CDCl_3_) and HMBC NMR spectra of taraxasterol acetate; Western blot images of IRS1, AKT, PI3K, AMPK, GLUT4, JNK and NF-κB proteins (Figures S1–S9) (PDF)
