# Supplementary material for: Taraxasterol Acetate Attenuates TNF-α-Induced Insulin Resistance via Regulation of Insulin Signaling, Inflammation, and Lipid Metabolism in 3T3-L1 Cells
Source: ACS Omega. 2026 Feb 26;11(9):15133–44. doi: 10.1021/acsomega.5c12241 (PMC12980172; doi:10.1021/acsomega.5c12241)
Supplement: Supplementary file 1 [file ao5c12241_si_001.pdf]

Supplementary Information

Taraxasterol Acetate Attenuates TNF-α-Induced Insulin Resistance via Regulation of Insulin Signaling, Inflammation, and Lipid Metabolism in 3T3-L1 Cells

Renan P. de Lima[#,*], Francisca Tuelly B. de Oliveira[*], Ana Virginia L. da Silva[*], Maria Rose Jane R. Albuquerque[†], Otília D. L. Pessoa[‡], Flávia A. Santos[*]

Department of Medicine, Weill Center for Metabolic Health, Cardiovascular Research Institute, Weill Cornell Medicine, New York, New York 10021, USA

* Department of Physiology and Pharmacology, Natural Products Laboratory, Faculty of Medicine, Federal University of Ceará, Fortaleza, Ceará 60430-270, Brazil

† Vale do Acaraú State University, Sobral, Ceará 62010-295, Brazil

‡ Department of Organic and Inorganic Chemistry, Sciences Center, Federal University of Ceará, Fortaleza, Ceará 60440-900, Brazil

Corresponding Author

Flávia A. Santos − Department of Physiology and Pharmacology, Natural Products Laboratory, Faculty of Medicine, Federal University of Ceará, 1315 Coronel Nunes de Melo, Rodolfo Teófilo, Fortaleza, 60430-270, Ceará, Brazil; Email: flavia@ufc.br

Contents

Figure S1. FT-IR spectrum of taraxasterol acetate.

Figure S2. MS (70 eV) of taraxasterol acetate.

Figure S3. ^{1}H NMR (500 MHz, $CDCl_3$) spectrum of taraxasterol acetate.

Figure S4. ^{13}C NMR (125 MHz, $CDCl_3$) spectrum of taraxasterol acetate.

Figure S5. HMBC NMR spectrum of taraxasterol acetate.

Figure S6: Western blot images corresponding to the data presented in Figure 3 of the main manuscript. Western blot analysis of IRS1 and AKT proteins.

Figure S7: Western blot images corresponding to the data presented in Figure 3 of the main manuscript. Western blot analysis of PI3K and AMPK proteins.

Figure S8: Western blot images corresponding to the data presented in Figure 3 of the main manuscript. Western blot analysis of GLUT4 protein.

Figure S9: Western blot images corresponding to the data presented in Figure 4 of the main manuscript. Western blot analysis of JNK protein.

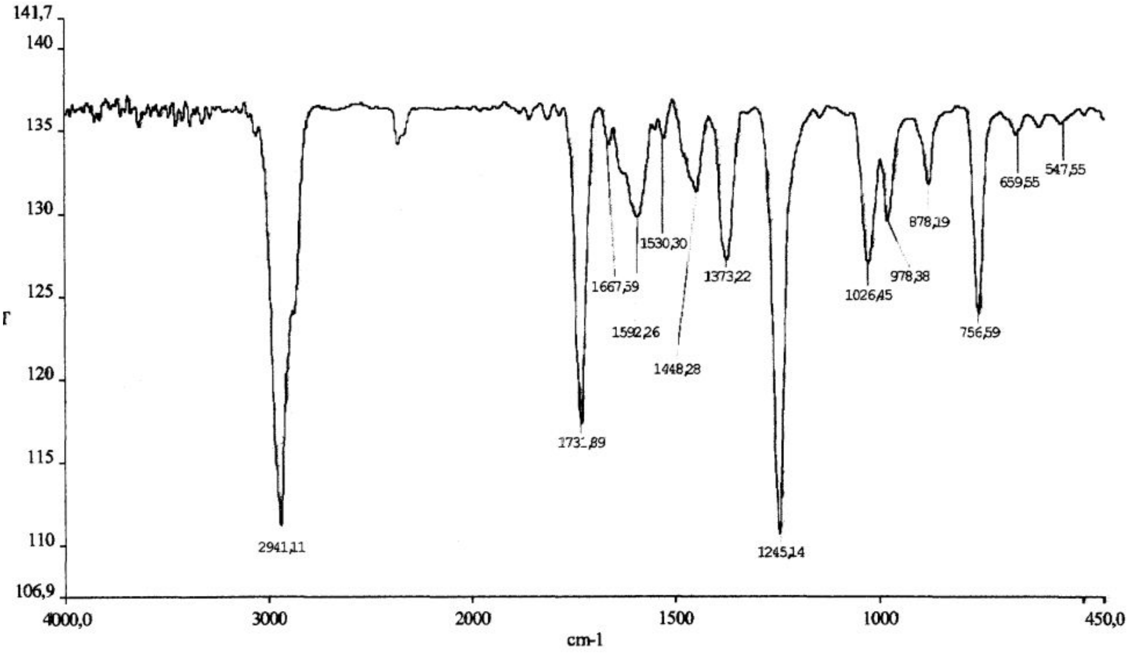

Figure S1. FT-IR spectrum of taraxasterol acetate.

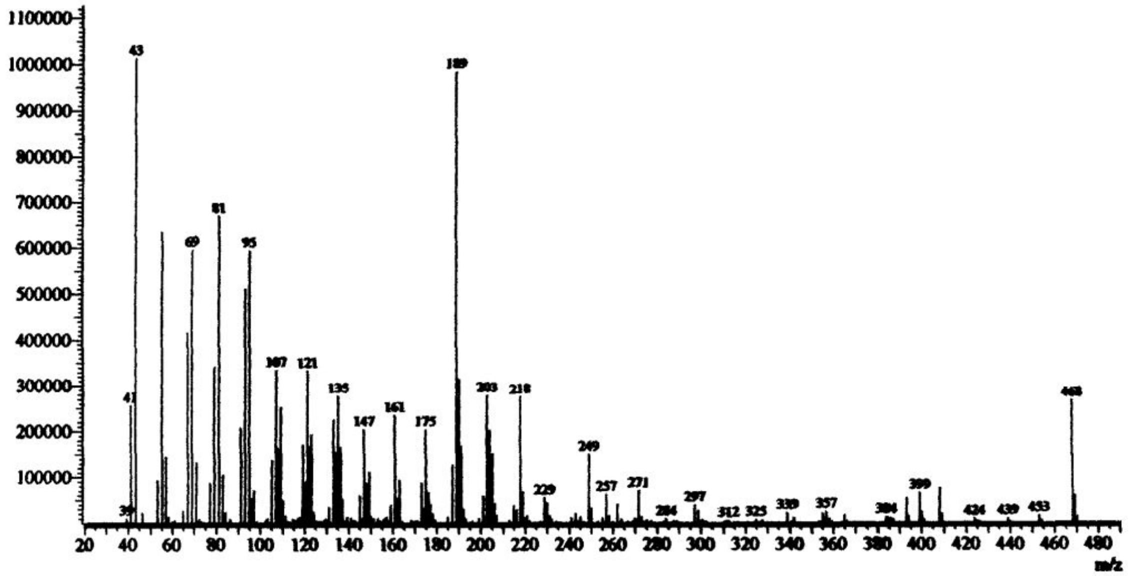

Figure S2. MS (70 eV) of taraxasterol acetate.

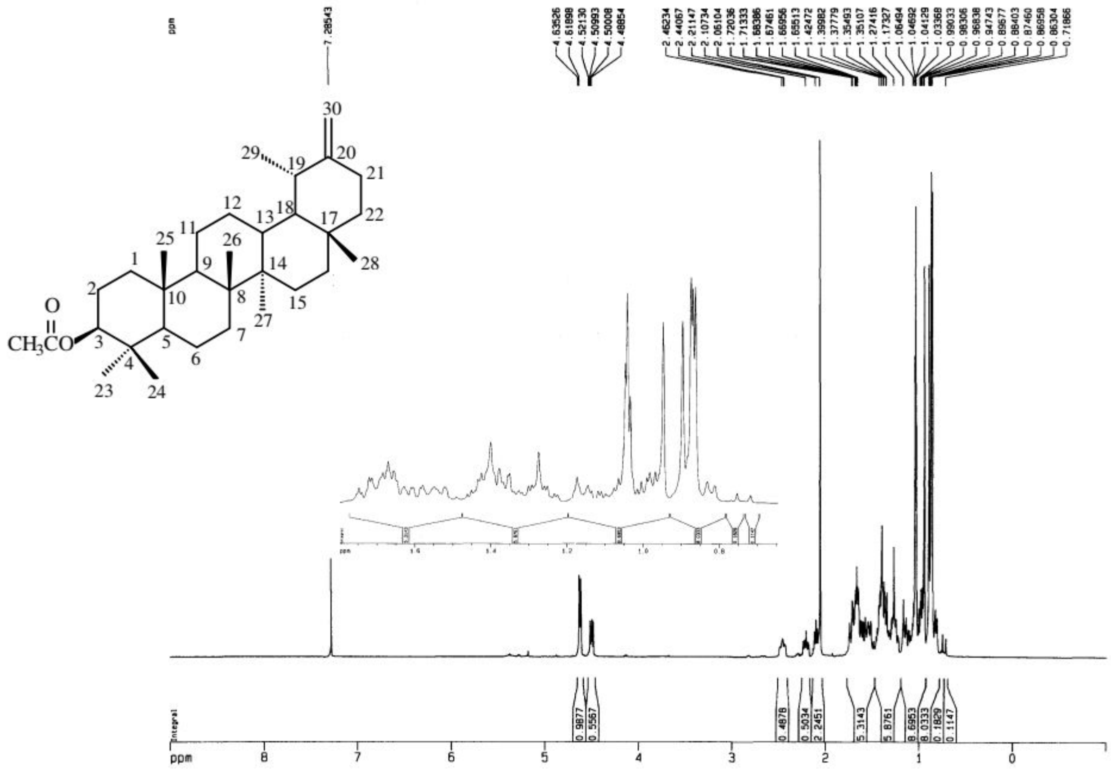

Figure S3. ^{1}H NMR spectrum (500 MHz, $CDCl_3$) of taraxasterol acetate.

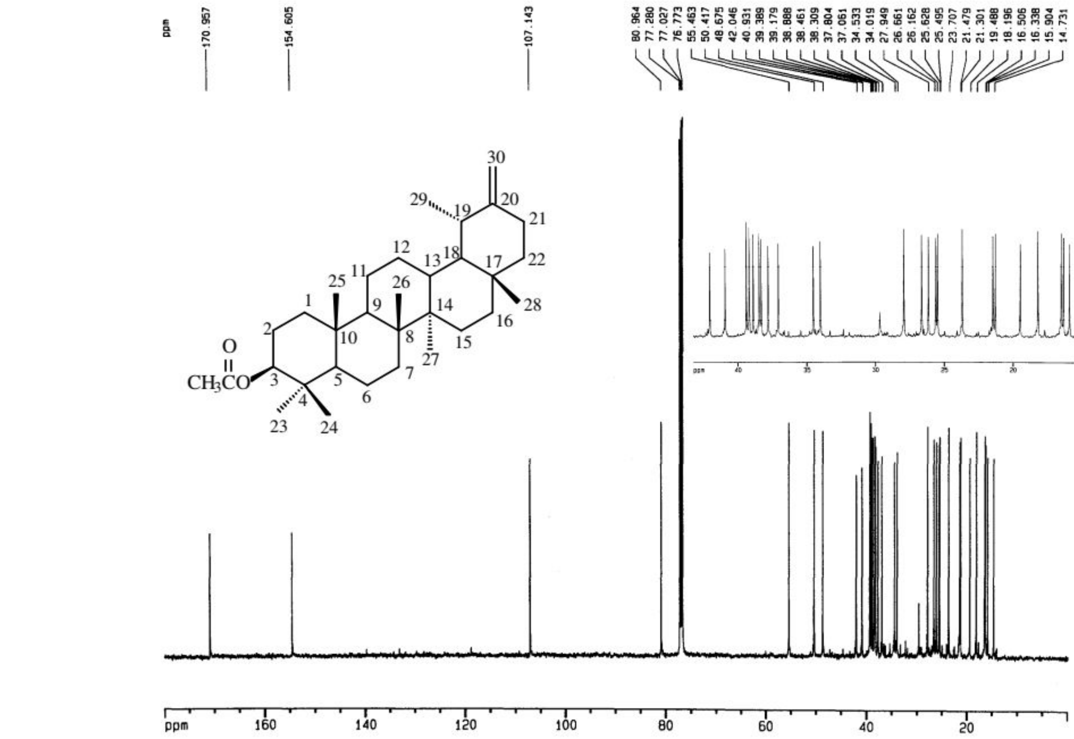

Figure S4. ^{13}C NMR spectrum (125 MHz, $CDCl_3$) of taraxasterol acetate.

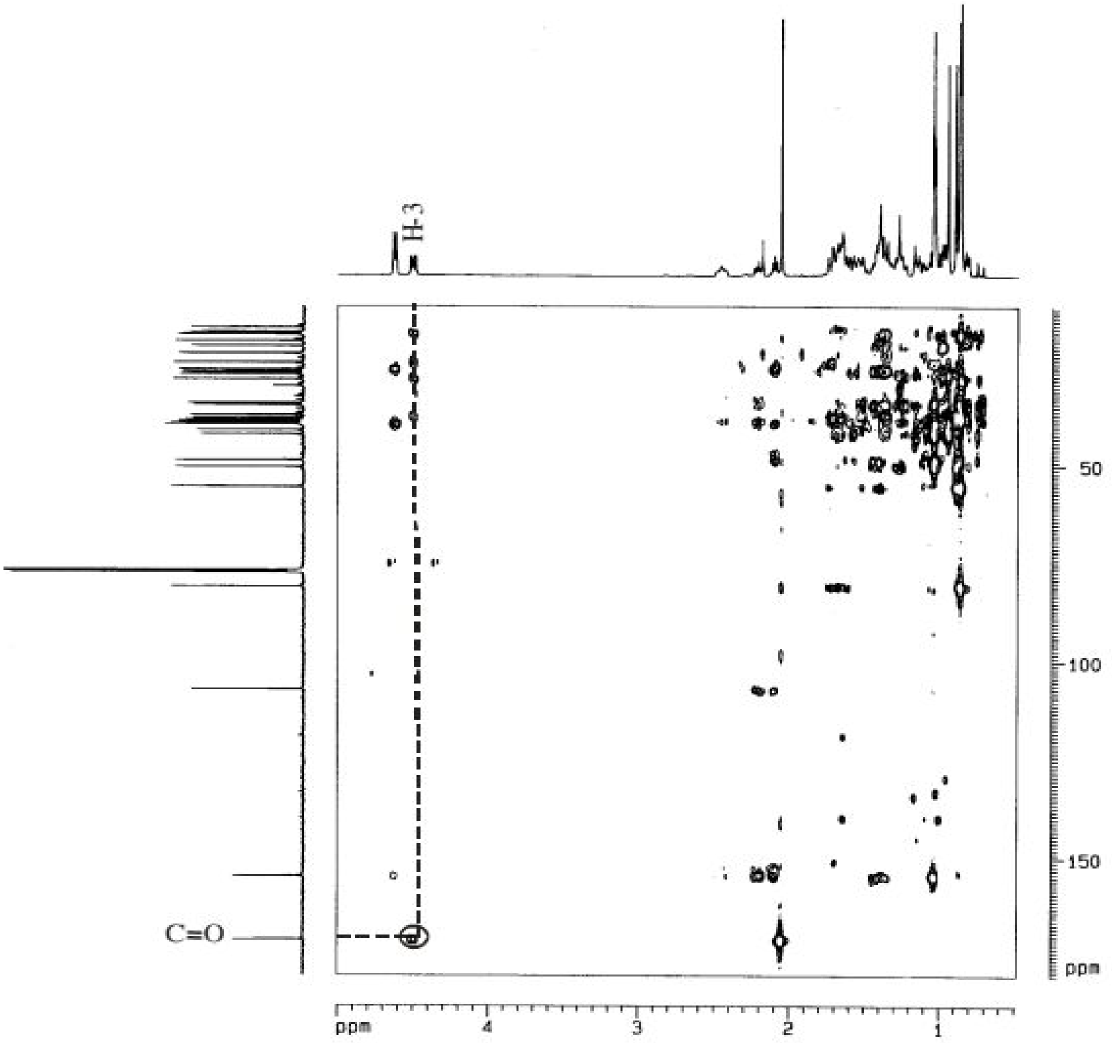

Figure S5. HMBC NMR spectrum of taraxasterol acetate.

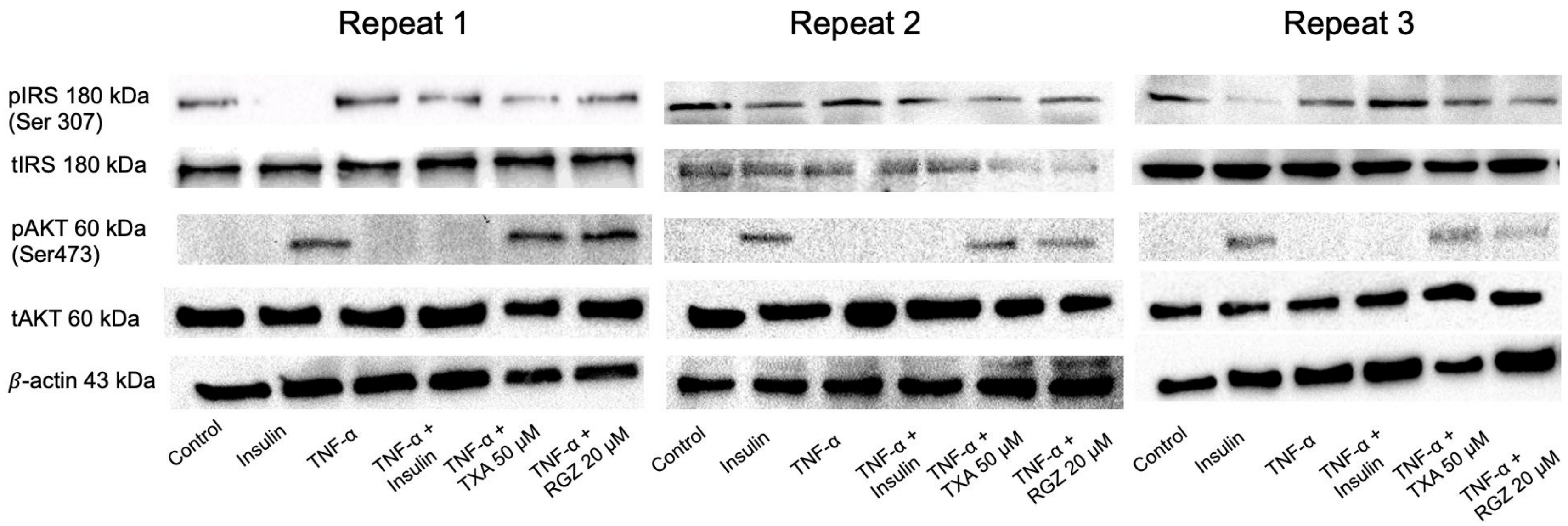

Figure S6. Western blot images corresponding to the data presented in Figure 3 of the main manuscript. Representative immunoblots of phosphorylated and total IRS1 (pIRS1 Ser307, tIRS1) and AKT (pAKT Ser473, tAKT) under the indicated treatment conditions. Cells were treated with insulin, TNF-α, or TNF-α in combination with insulin, TXA (50 μM), or RGZ (20 μM). β-actin was used as a loading control. Three independent biological replicates are shown.

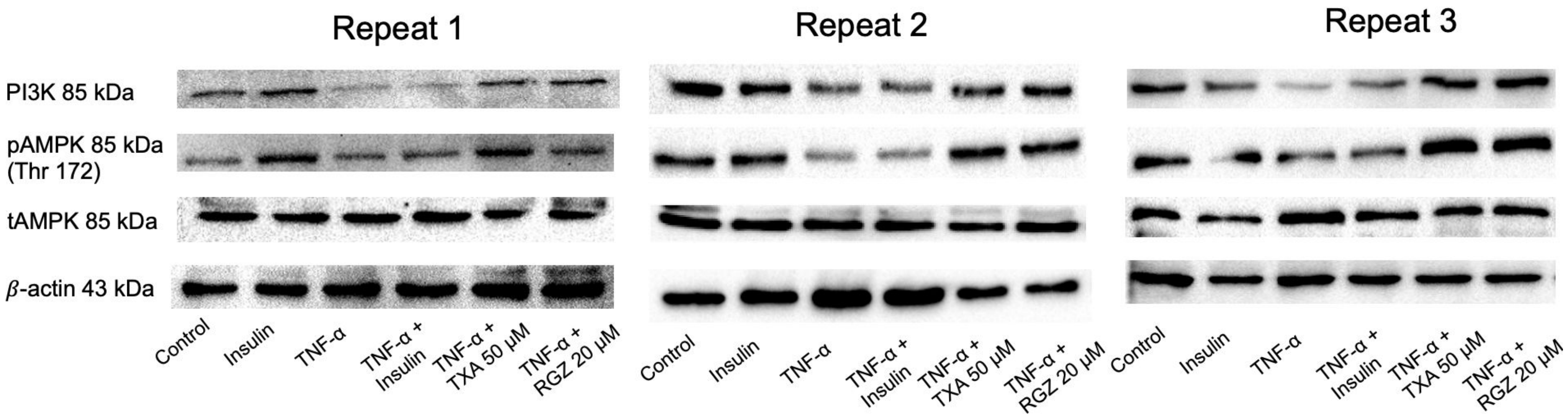

Figure S7. Western blot images corresponding to the data presented in Figure 3 of the main manuscript. Representative immunoblots of PI3K and phosphorylated and total AMPK (pAMPK Thr172, tAMPK) under the indicated treatment conditions. Cells were treated with insulin, TNF-α, or TNF-α in combination with insulin, TXA (50 μM), or RGZ (20 μM). β-actin was used as a loading control. Three independent biological replicates are shown.

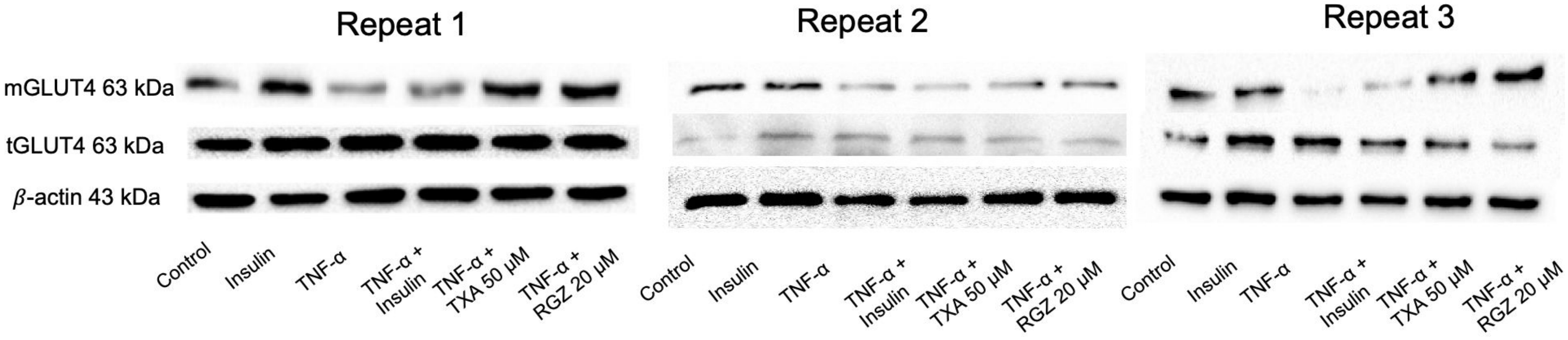

Figure S8. Western blot images corresponding to the data presented in Figure 3 of the main manuscript. Representative immunoblots of membrane-associated and total GLUT4 (mGLUT4, tGLUT4) under the indicated treatment conditions. Cells were treated with insulin, TNF-α, or TNF-α in combination with insulin, TXA (50 μM), or RGZ (20 μM). β-actin was used as a loading control. Three independent biological replicates are shown.

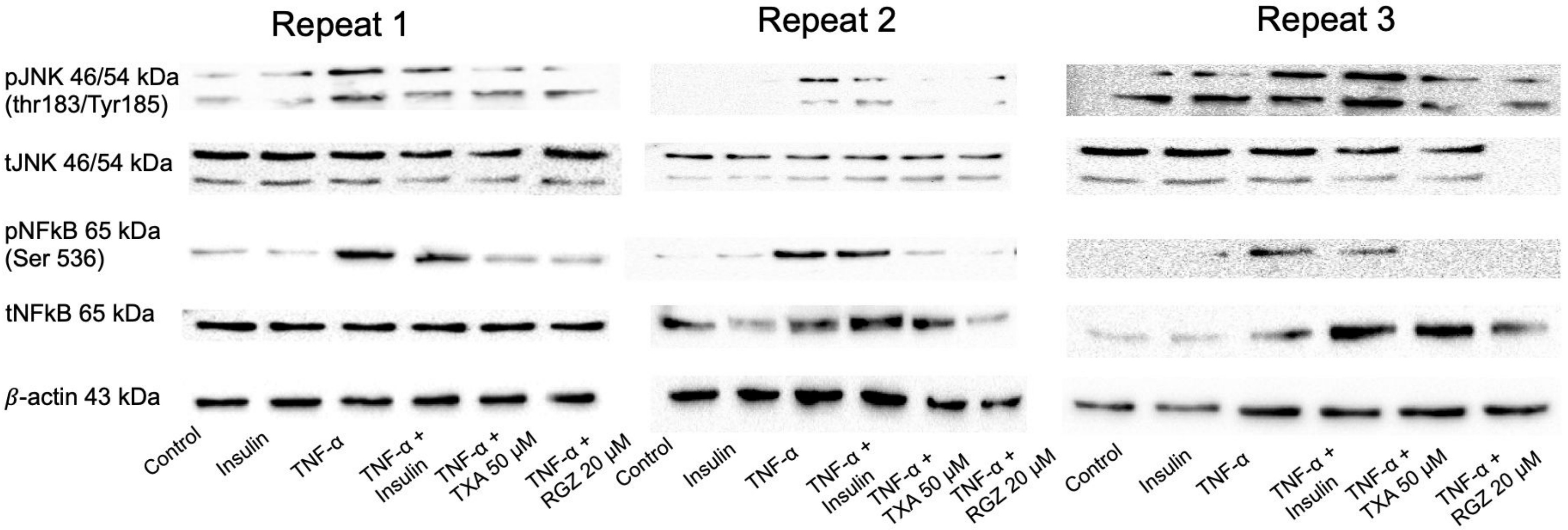

Figure S9. Western blot images corresponding to the data presented in Figure 4 of the main manuscript. Representative immunoblots of phosphorylated and total JNK (pJNK Thr183/Tyr185, tJNK 46/54 kDa) and NF-κB p65 (pNF-κB Ser536, tNF-κB) under the indicated treatment conditions. Cells were treated with insulin, TNF-α, or TNF-α in combination with insulin, TXA (50 μM), or RGZ (20 μM). β-actin was used as a loading control. Three independent biological replicates are shown.
